# Hydatid Disease Located in the Cerebellomedullary Cistern

**DOI:** 10.1155/2014/271365

**Published:** 2014-03-24

**Authors:** Özgür Kızılca, Murat Altaş, Utku Şenol, Murat Alp Öztek

**Affiliations:** ^1^Department of Radiology, Akdeniz University Faculty of Medicine, 07058 Antalya, Turkey; ^2^Department of Neurosurgery, Akdeniz University Faculty of Medicine, 07058 Antalya, Turkey

## Abstract

Hydatid disease is an endemic zoonotic disease in many areas of the world. Liver, followed by lung, is the most commonly affected organ and involvement of other organs is rare. When brain is involved, lesions are typically supratentorial, and infratentorial localisation is even rarer. We present a 45-year-old woman with hydatid disease located in premedullary location compressing the brain stem, an exceedingly rare location for cerebral echinococcosis. Relevant literature regarding typical properties of cerebral disease was reviewed.

## 1. Introduction

Hydatid disease is endemic in many areas, especially in the Middle East, Turkey, South America, South Europe, New Zealand, and Australia [1–3]. The patients are usually asymptomatic or their symptoms are nonspecific since growth of the cysts is generally slow and therefore clinical manifestations tend to be nonspecific complaints due to compression of involved organs [2, 4]. The diagnosis depends on clinical suspicion, typically based on a history of living in, or having travelled to, an endemic area and contact with cattle or dogs and is confirmed with serologic tests and imaging [3].

The most common locations for hydatid cysts are the liver, followed by the lung [1, 2]. However, many parts of the body can be affected, including bones, pericardium, orbits, and brain [1, 5]. Cerebral localization is extremely rare, being seen in 2-3% of systemic disease and forming 2% of all intracranial space occupying lesions [2, 3, 5]. This rarity, coupled with nonspecific symptoms, necessitates a high degree of clinical suspicion and thus presents a diagnostic difficulty. We present a case of infratentorial cerebral hydatid disease with an exceedingly rare location, followed by a review of the literature regarding typical characteristics and imaging findings of cerebral echinococcosis.

## 2. Case

**Figure 1:**
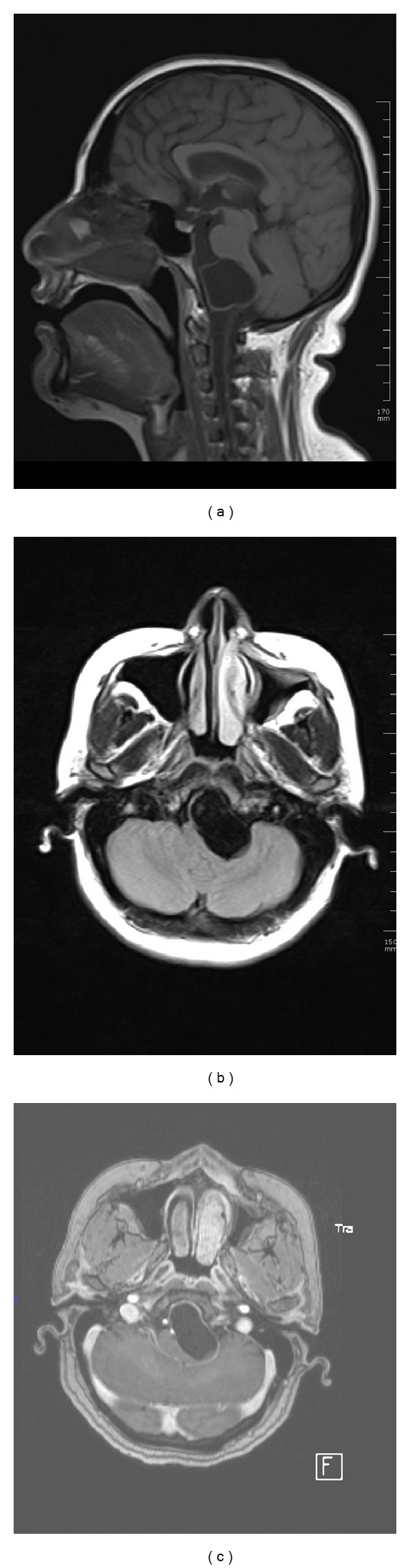
(a) T1W sagittal, (b) FLAIR transverse, and (c) postcontrast T1W transverse images. An unhanced cystic mass is seen in the premedullary space. The slightly left parasagittally located mass is compressing the brainstem left anteriorly.

A 45-year-old woman applied to a different center with complaints of nausea, vomiting, and headache that has been going on for three months. Following tests and examination, she was referred to our center with the initial diagnosis of a mass. Her physical examination revealed monoparesis in the left lower extremity and hypoesthesia on the left but was otherwise normal. Magnetic resonance imaging (MRI) in our center revealed an extra-axially located cystic lesion with a thin wall in the premedullary location compressing brain stem. The patient was operated and the lesion was revealed to be hydatid cyst (Figure 1).

## 3. Discussion

Hydatid disease, also called echinococcosis, is a parasitic disease caused by larvas of* Echinococcus* tapeworms [1].* E. granulosus* and* E. multilocularis* are responsible for the majority of human disease, the former being more common. Even though places where the disease is endemic, namely, the Middle East, Turkey, Central and Southern Europe, Australia, South America, and New Zealand, are widely known, it should be kept in mind as a differential diagnosis even in nonendemic areas due to the ease of travel and migration from endemic to nonendemic places [1–3, 6]. Liver is the most commonly affected organ (50–77%), followed by the lung (8.5–43%) [1, 2]. Even though rare compared to the involvement of these two organs, many other tissues such as muscle, heart, kidney, bone, eye, skin, and spinal cord can also be affected [1, 5].

Cerebral involvement is seen in 2-3% of cases with systemic disease and is a rare cause of intracerebral space occupying lesions even in endemic areas. It is observed more often in children and young adults and several case series reported a slight male prevalence [7–10]. Patients may have hydatid cysts in other organs, with studies reporting extracerebral disease in 6–70% of patients [2, 4, 10]. They can be seen in any part of the brain but are usually supratentorial and located in the middle cerebral artery territory, most commonly the parietal lobe [2, 3, 11]. Sometimes large, single cysts can be observed in the frontoparietotemporal region [11, 12]. Infratentorial localization is exceedingly rare, and intraventricular, pontine, meningeal, cerebellar, intrasellar, cavernous sinus, aqueduct of Sylvius, and skull cysts, despite being even rarer, have been reported [3].

In the brain,* E. granulosus* caused cerebral cystic echinococcosis, where lesions are usually single and occurrence of multiple cysts is very rare. Cerebral alveolar echinococcosis, caused by* E. multilocularis*, may have single or multiple cysts but is much rarer compared to cystic disease [3]. The signs and symptoms are nonspecific and the most common ones are reported to be headache, papilledema, and vomiting; however, any symptom due to increased intracranial pressure can be seen [2, 3, 8, 10–13]. Focal symptoms like hemiparesis, seizures, gait, or sight disorders can be observed depending on the size and location of the lesion [10, 11].

In cerebral cystic echinococcosis both computerized tomography (CT) and MRI reveal a spherical cystic lesion with well-defined borders, a smooth thin wall with or without septae or calcification. The cyst wall is iso- or hyperdense with respect to the cerebral parenchyma on unenhanced CT and usually shows a rim of low intensity in both T1W and T2W images. Daughter cysts, if present, are considered pathognomonic but are rarely seen [2, 7]. Wall calcification is seen in less than 1% of cases [3, 7]. Mass effect, with compression of midline structures and the ventricles, is a common finding, but surrounding edema and rim enhancement are generally not seen in uncomplicated cases [2, 3, 14]. The imaging findings of alveolar echinococcosis include a solid, semisolid, or multiloculated cystic mass with well-defined margins. In contrast to cystic disease, calcification, edema in the surrounding tissues, and contrast enhancement in the inflammation region around the lesion are common [3].

The differential diagnosis of an intracerebral hydatid cyst with mainly typical characteristics includes supratentorial cystic lesions like arachnoid cysts, cystic tumors, abscess, and porencephalic cysts [2, 3, 5]. Arachnoid cysts are not spherical, porencephalic cysts are usually connected to the ventricular system and neither are entirely surrounded by brain tissue, cystic tumors usually have soft tissue components that are enhanced after contrast injection, and abscesses typically demonstrate enhancement and perifocal edema [2, 3, 5]. When the location is atypical, such as an infratentorial location as in our case, cystic lesions of the involved region, for example, neurenteric cysts for posterior fossa lesions, are first considered in the differential diagnosis. However, hydatid disease should be kept in mind in the setting of appropriate medical history.

MR spectroscopy has potential, albeit experimental, application in diagnosing cerebral hydatid disease. In a series, three cases of hydatid cysts have been reported to show lactate, acetate, and succinate peaks, and one case with surrounding edema revealed a choline and mannitol peak. Arachnoid cysts, on the other hand, showed only small lactate peaks and tumoral lesions demonstrated increases in choline and lactate with decreased NAA [15].

In conclusion, imaging is useful, but not always conclusive, in diagnosing cerebral hydatid disease. CT detects calcification in the lesion better than MRI, whereas MRI is superior in defining the exact location and anatomic relationships of the lesion [2, 3, 11]. Nevertheless, in some cases, despite use of advanced imaging techniques, the diagnosis remains problematic [11]. Hydatid disease should be considered especially with supratentorial cystic lesions in young male patients with a history of living in an endemic area or having contact with cattle or dogs; however, as in our case, atypical cases of infratentorial localization in an older woman are possible and, thus, the differential diagnosis for any cystic lesion of the brain in a patient with appropriate medical history should include echinococcosis.
